# Clinicopathologic and Molecular Characterization of SMARCB1-Deficient Sinonasal Carcinomas – A Systematic Study from a Single Institution Cohort

**DOI:** 10.1007/s12105-025-01788-w

**Published:** 2025-05-14

**Authors:** Qinyuan Li, Tarek Abi-Saab, Andrey Prilutskiy, Vanessa Horner, Leah Frater-Rubsam, Yajing Peng, Wei Huang, Randall J. Kimple, Paul M. Harari, Ricardo V. Lloyd, Rong Hu

**Affiliations:** 1 Department of Pathology and Laboratory Medicine, University of Wisconsin-Madison, Madison, WI 53705 USA; 2 Wisconsin State Laboratory of Hygiene, Madison, WI 53706 USA; 3 Department of Human Oncology, University of Wisconsin-Madison, Madison, WI 53705 USA; 4 McArdle Laboratory for Cancer Research, Madison, WI 53705 USA

**Keywords:** SWI/SNF complex, SMARCB1, SMARCA4, Sinonasal carcinoma, EWSR1

## Abstract

**Background:**

SMARCB1-deficient and SMARCA4-deficient sinonasal carcinomas are rare, with only a few systematic studies available in the literature. Secondary *EWSR1* gene abnormalities have been reported in SMARCB1-deficient tumors. This study aimed to systematically investigate SWI/SNF complex-deficient sinonasal carcinomas in a single-institution cohort, perform clinicopathologic characterization, and explore the underlying molecular mechanisms.

**Method:**

Immunohistochemistry (IHC) of INI1 and BRG1 was performed on tissue microarrays containing tumor tissue from 149 consecutive sinonasal carcinomas. Single nucleotide polymorphism (SNP) array and *EWSR1* gene fluorescence in situ hybridization (FISH) analyses were conducted on SMARCB1-deficient sinonasal carcinomas. Clinicopathologic characterization was studied.

**Result:**

Of the 149 sinonasal carcinomas, 7 (4.7%) showed SMARCB1 loss, while none demonstrated SMARCA4 loss. All patients were male and presented with advanced-stage tumors. Four SMARCB1-deficient sinonasal carcinomas exhibited basaloid morphology, two displayed eosinophilic tumor morphology, and one had mixed morphology. Homozygous and heterozygous *SMARCB1* deletions were identified in 4/6 and 2/6 cases respectively. Heterozygous loss involving genes neighboring *SMARCB1 gene*, including *EWSR1*, was observed in four cases. One tumor showed a heterozygous loss of the entire chromosome 22q. *EWSR1* FISH assay revealed concordant heterozygous *EWSR1* loss in these five cases.

**Conclusion:**

SMARCB1-deficient carcinomas account for 4.7% of sinonasal carcinomas in this single-institution cohort, while SMARCA4-deficient tumors are even rarer, with none identified. SMARCB1-deficient sinonasal carcinomas exhibit a broad spectrum of morphologic and immunohistochemical features. These carcinomas show complex genetic alterations, with homozygous *SMARCB1* deletions present in the majority of cases.

**Supplementary Information:**

The online version contains supplementary material available at 10.1007/s12105-025-01788-w.

## Introduction

The SWItch/Sucrose NonFermentable (SWI/SNF) complex is crucial in the chromatin remodeling process and plays important roles in the regulation of gene expression, cell proliferation, and differentiation [1–3]. This complex is composed of more than 20 subunits; inactivation of these subunits through genetic mutations or epigenetic silencing disrupts chromatin remodeling and has been increasingly recognized as a molecular driver for a variety of human neoplasms across different histogenetic lineages [4–9]. Among the subunits that have been studied extensively (SMARCB1, SMARCA4, SMARCA2, and ARID1A), the loss of SMARCB1 and SMARCA4 has been implicated in sinonasal carcinomas. SWI/SNF complex-deficient sinonasal carcinoma, defined by the loss of SMARCB1 or SMARCA4, was recently recognized as a distinct entity in the 5th edition of the World Health Organization (WHO) Classification of Head and Neck Tumors, highlighting its unique clinicopathologic and molecular profile [10].

SMARCB1-deficient sinonasal carcinoma is rare, with fewer than 200 cases reported in the literature since its discovery in 2014 [11–13]. SMARCA4-deficient sinonasal carcinoma is even rarer, with fewer than 22 cases reported since the first report in 2017 [14, 15]. Many of the studies are case reports, small case series, or multi-institutional pooled cohort studies, with only few systematic studies performed [12, 16, 17]. SWI/SNF-deficient sinonasal carcinoma is often poorly differentiated in morphology. Many cases may have been misclassified as other sinonasal carcinoma subtypes in the past and could still be misclassified nowadays. As a result, the true frequency of these tumors is not well established due to the scarcity of systematic studies and the rarity of the tumors themselves.

Somatic *SMARCB1* deletions have been found in many SMARCB1-deficient tumors. The incidence of *SMARCB1* gene deletions varies depending on the tumor type and anatomical location. Furthermore, the region involved on chromosome 22q11-12 shows significant diversity and complexity [18, 19]. Studies on the molecular underpinnings of SMARCB1 protein loss, using a variety of testing tools such as fluorescence in situ hybridization (FISH) and next-generation sequencing, have demonstrated homozygous (biallelic) or heterozygous (monoallelic) deletions of the *SMARCB1* gene in SMARCB1-deficient sinonasal carcinomas [12, 17, 20, 21].

The *SMARCB1* and *EWSR1* genes are located on chromosomes 22q11.23 and 22q12.2, respectively, approximately 5.5 megabases apart. *EWSR1* gene alterations may occur secondary to *SMARCB1* gene deletion. Cases of SMARCB1-deficient tumors, such as extrarenal rhabdoid tumors and myoepithelial carcinomas, harboring concurrent *EWSR1* gene abnormalities, which resulted in misinterpretation of *EWSR1* gene FISH results, have been reported [22].

This study aimed to systematically investigate SWI/SNF complex-deficient sinonasal tract carcinomas in a single-institution cohort, perform clinicopathologic characterization, and explore the underlying molecular mechanisms of *SMARCB1* and *EWSR1* gene abnormalities in SMACRB1-deficient sinonasal carcinomas.

## Materials and Methods

### Sinonasal Carcinoma Tissue Microarray (TMA)

Tissue microarrays (TMAs) were constructed from formalin-fixed, paraffin-embedded (FFPE) tissue blocks of 149 consecutive sinonasal carcinoma cases diagnosed at the University of Wisconsin–Madison between January 1991 and June 2023, as previously described [23]. Each case was individually confirmed as being primary to the sinonasal tract. Triplicate 1-mm cores from each case were included in the TMA. Tumors were classified according to the 5th edition of the WHO Tumor Classification [10]. Relevant clinical information was obtained through reviewing of electronic medical records. This study was conducted in accordance with our institution’s IRB approval (Approval No. 2018 − 1510).

### Immunohistochemical Staining and High-Risk HPV E6/7 in Situ Hybridization

Immunostaining for INI1 (clone MRQ-27, prediluted, Cell Marque, Rocklin, CA), BRG1 (clone EONCIR111A, 1:100 dilution, Abcam, Cambridge, UK), NUT (clone PA5-59171, 1:100 dilution, Thermo Fisher Scientific, Waltham, MA), and p16 (clone E6H4™, prediluted, Ventana Medical Systems, Inc., Tucson, AZ) was performed on TMA sections using the Ventana Benchmark Ultra (Roche Diagnostics), following the manufacturer’s automated protocols. Protein expression was visualized using 3,3’-diaminobenzidine (DAB). For cases demonstrating loss of INI1 or BRG1 expression on TMAs, additional immunostaining was performed on whole tissue sections for confirmation, if not previously done during the original case workup. Antibodies used in the immunohistochemical staining performed during the initial workup was listed in the supplemental Table 1.

In situ hybridization (ISH) for high-risk HPV E6/E7 mRNA was performed on TMA sections manually using the RNAscope kit, which targets 18 high-risk genotypes (HPV 16, 18, 26, 31, 33, 35, 39, 45, 51, 52, 53, 56, 58, 59, 66, 68, 73, and 82; Advanced Cell Diagnostics, Inc., Hayward, CA), as described previously [24].

### Single Nucleotide Polymorphism Array Analysis (SNP) Targeting SMARCB1 and EWSR1 (22q11.23q12.2)

All SMARCB1-deficient sinonasal carcinomas were tested for *SMARCB1* and *EWSR1* gene copy number variants and regions of homozygosity by SNP array analysis [25]. This array assay included 1.8 million distinct locus-specific probes with at least 15x redundancy. The probes were spaced an average of 20 kilobases (kb) apart across the entire genome (backbone coverage), with increased probe density (5 kb) in targeted clinically relevant genes.

Briefly, genomic DNA was extracted from formalin-fixed, paraffin-embedded (FFPE) tissue sections. Approximately 2 mm³ of FFPE tumor tissue was meticulously scraped from slides using a sterile razor blade. DNA extraction was performed using the Maxwell^®^ RSC DNA FFPE Kit and Maxwell^®^ Instrument (Promega, USA), following the manufacturer’s guidelines. The quality of the extracted DNA was assessed using the Nanodrop One (Thermo Fisher Scientific, Madison, WI, USA) and the Genomic DNA ScreenTape Assay (Agilent, USA). Quantification of double-stranded DNA (dsDNA) was carried out using the Qubit^®^ 2.0 fluorometer (Invitrogen, USA).

Subsequently, SNP array analysis was performed using the Infinium Global Diversity Array with Cytogenetics-8 kit (Illumina, USA), following standard protocols. Image capture was performed with an iScan System (Illumina, USA) to generate IDAT files. These files were converted to GTC files using Beeline 2.0 software (Illumina, USA) with an FFPE-specific cluster file. Image analysis and automated Copy Number Variation (CNV) calling for the 22q11.23q12.2 region were carried out using VIA™ Analysis Software (Bionano, USA).

### EWSR1 Gene Fluorescence in Situ Hybridization (FISH)

*EWSR1* gene FISH assay using the Vysis LSI EWSR1 Dual Color Break Apart Rearrangement FISH Probe Kit (Abbott Molecular, Abbott Park, IL) was performed on all SMARCB1-deficient sinonasal carcinomas. This probe kit contains a mixture of 2 FISH DNA probes. The first probe, a 497 kb probe labeled in Spectrum Orange, flanks the 5’ side of the *EWSR1* gene, and extends inward into intron 4. The second probe, a 1100 kb probe labeled in Spectrum Green, flanks the 3’ side of the *EWSR1* gene. The known break points within the *EWSR1* gene are restricted to introns 7 through 10.

Briefly, FFPE tissue sections were deparaffinized in Citrisolv Hybrid (Decon Labs, King of Prussia, PA), followed by sequential treatment with 0.2 N HCl, 1 M sodium thiocyanate, Protease I (Abbott Molecular), 10% formalin, and a dehydrating ethanol series (70%, 85%, and 95%). Tissue and probe were codenatured by heating at 80°C for 2 minutes using a ThermoBrite instrument (Abbott Molecular). Hybridization was performed overnight at 37°C in IntelliFISH hybridization buffer (Abbott Molecular). After hybridization, the slides were mounted with Vectashield containing DAPI (Vector Laboratories, Burlingame, CA). The fluorescence signals were analyzed for 200 interphase nuclei per region of interest identified by a pathologist (RH). Heterozygous deletion of the *EWSR1* gene was defined as the presence of only one copy of the gene (detected by both the 5’ and 3’ end probes) without break apart in more than 25% of the nuclei.

## Results

### Frequency of the SWI/SNF Complex - Deficient Sinonasal Carcinoma

Among the 149 sinonasal carcinomas analyzed, SMARCB1 (INI1) expression was lost in 7 tumors (4.7%) (95% confidence interval: 1.7–10.3%), while none showed loss of SMARCA4 (BRG1) expression by immunohistochemical staining (0/149) (95% confidence interval: 0 to 4.6%). Of the 7 SMARCB1-deficient tumors, 4 were correctly diagnosed prospectively, while 3 archived cases were retrospectively reclassified during this study. The original diagnoses for these 3 cases, rendered between 1999 and 2014, included poorly differentiated carcinoma (*n* = 1), nonkeratinizing squamous cell carcinoma (*n* = 1), and poorly differentiated adenosquamous cell carcinoma (*n* = 1).

The remaining 142 sinonasal carcinomas were classified as follows: 19 salivary gland carcinomas, 91 squamous cell carcinomas, 7 adenocarcinomas, 3 NUT carcinomas, 1 HPV- associated multiphenotypic sinonasal carcinoma, 19 poorly/undifferentiated carcinomas, 1 low grade sinonasal papillary carcinoma and 1 large cell neuroendocrine carcinoma.

### Clinicopathological Features of the SMARCB1-Deficient Sinonasal Carcinoma

The clinicopathological characteristics of SMARCB1-deficient sinonasal carcinomas are summarized in Table 1. All patients were male, with ages ranging from 34 to 74 years (median age of 60). The entire sinonasal carcinoma cohort included 108 male and 41 female patients, with a median age of 62 years. Four tumors were primarily located in the maxillary sinus, and three were from the ethmoid sinus. Six tumors were staged T4, and one tumor was staged T3.

**Table 1:** Clinicopathologic characteristics of the SMARCB1-deficient sinonasal carcinoma (*n* = 7)

Case	Pt Age	Pt Sex	Tumor Site	Tumor Morphology	Clinical Stage	Treatment	Patient Outcome
1	55	M	Maxillary Sinus	Basaloid	T4N0M0	Surgery + Radiation	Recurrence with bone mets in 11 mos, DOD in 20 mos
2	60	M	Maxillary Sinus	Basaloid	T4N0M0	Surgery + Chemoradiation	NED (FU 50 mos)
3	71	M	Maxillary Sinus	Basaloid	T3N0M0	Surgery + Radiation	NED (FU 87 mos)
4	59	M	Ethmoid Sinus	Basaloid	T4N0M0	Chemoradiation	Disease progression with lung mets in 18 mos; LOF (FU 21 mos)
5	69	M	Ethmoid Sinus	Mixed with predominant basaloid	T4N0M0	Chemoradiation	Local recurrence with Lung mets in 26 mos, LOF (FU 27 mos)
6	74	M	Maxillary Sinus	Eosinophilic	T4N0M0	Surgery + Chemoradiation	LN and lung Mets in 5 mos; DOD in 6 mos
7	34	M	Ethmoid	Eosinophilic	T4N2M1	Chemoradiation	DOD in 5 mos

Pt, patient; M, Male; mets; metastasis; Mos, months; DOD, died of disease; NED, no evidence of disease; FU, follow-up; LOF, loss of follow-up; LN, lymph node

Four patients underwent surgical resection followed by adjuvant chemoradiation therapy, while three patients received chemoradiation alone. The follow-up period ranged from 5 to 87 months, with a median follow-up of 21 months. Three patients died of the disease at 5-, 6-, and 20-months post-treatment, respectively. Two patients experienced disease progression and distant metastasis at 18- and 26- months post-treatment, respectively, but were lost to follow-up thereafter. Two patients have remained disease-free since the treatment (surgery followed by chemoradiation therapy), with follow-up durations of 50 and 87 months, respectively. There was no notable clinicopathologic difference between the two long-term survivors and the other patients.

### Histopathologic Features of the SMARCB1-Deficient Sinonasal Carcinoma

**Fig. 1:**
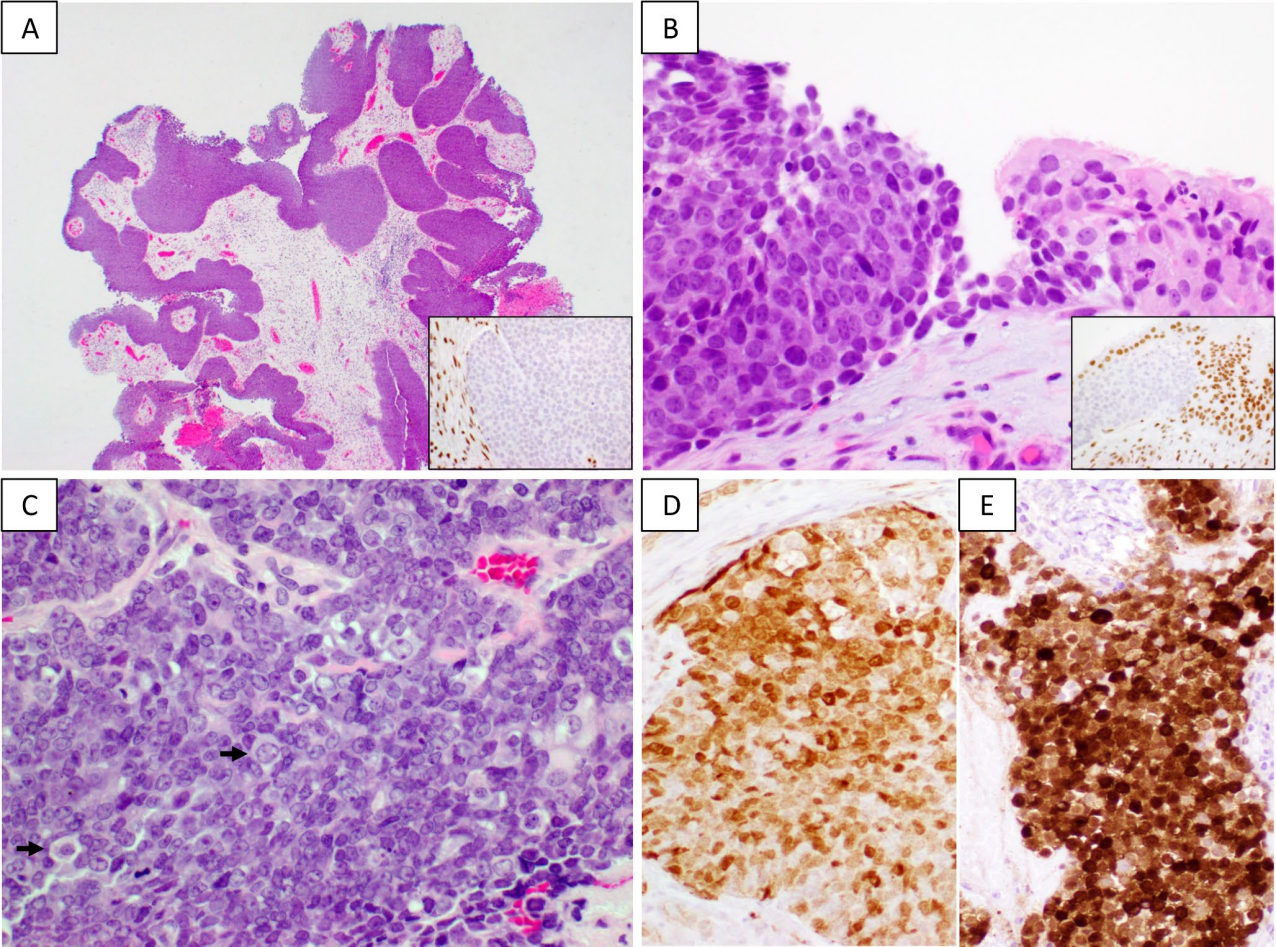
Representative images of basaloid SMARCB1-deficient sinonasal carcinomas. **A**. The tumor exhibits exophytic/papillary growth, lined by basaloid tumor cells (200x). **B**. Tumor cells colonize the sinonasal mucosa surface, transitioning to adjacent benign sinonasal epithelium (400x). Panels A and B are images from case 2. **C**. The tumor cells are monotonous, with occasional plasmacytoid tumor cells (arrows) scattered throughout the tumor (case 3) (200x). **D** and **E**. Aberrant p16 overexpression with predominantly nuclear staining in two basaloid tumors (case 2 and case 4 respectively). The insets in A and B show loss of INI1 expression in tumor cells and retained INI1 expression in adjacent stromal cells and benign sinonasal epithelium

Four SMARCB1-deficient sinonasal carcinomas exhibited a basaloid (“blue tumor”) appearance (Table 1), morphologically resembling nonkeratinizing squamous cell carcinoma (SCC) of the sinonasal tract. Three of these tumors showed exophytic or papillary growth, lined by basaloid tumor cells (Fig. 1A). The tumor also exhibited inverted growth patterns, forming large nests or broad bands within the stroma, with or without associated inflammatory infiltrates. Comedonecrosis was observed in two cases. Stromal desmoplasia was uncommon. Tumor colonization on the mucosal surface, transitioning into benign sinonasal epithelium, was also noted (Fig. 1B). The tumor cells were monotonous, with a high nuclear-to-cytoplasmic (N/C) ratio, and demonstrated frequent mitoses and apoptosis. Cells with a plasmacytoid appearance were scattered throughout the basaloid tumor under high-power magnification (Fig. 1C).

**Fig. 2:**
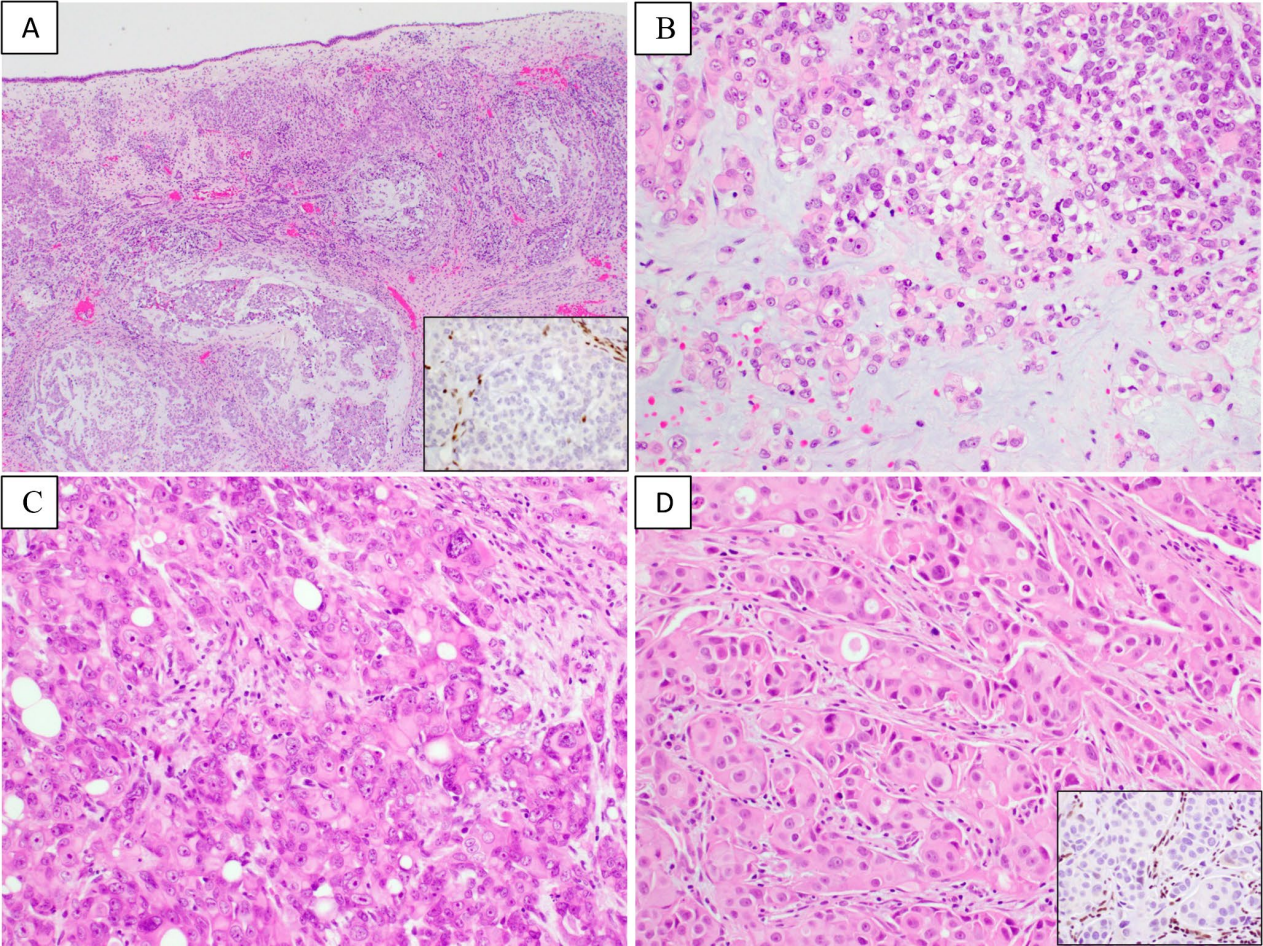
Representative images of eosinophilic SMARCB1-deficient sinonasal carcinomas. **A**. Surface of the tumor is lined by intact sinonasal epithelium without exophytic growth. Tumor infiltrates the stroma, with associated desmoplastic changes. A myxoid matrix is present in this case (40x). **B**. The tumor cells contain abundant eosinophilic cytoplasm, intermixed with cells exhibiting clear cytoplasm (200x). **C**. Focal nuclear anaplasia is observed (200x). Panels A, B and C are images from case 6. **D**. This eosinophilic tumor (case 7) exhibits occasional lumina without frank glandular differentiation (200x). The insets in A and D show loss of INI1 expression in tumor cells and retained INI1 expression in adjacent stromal cells

Two tumors manifested as overly eosinophilic tumor (“pink tumor”) (Table 1) upon low-power microscopic review (Fig. 2A). Exophytic growth was not observed in these tumors. The tumor infiltrated the stroma, forming large and small nests with evident stromal desmoplasia (Fig. 2A). The tumor cells exhibited eosinophilic/pink cytoplasm, and plasmacytoid morphology was frequently observed (Fig. 2B). The tumor cells were mostly monotonous, with focal nuclear anaplasia present in one case, including cells with giant nuclei and multinucleated tumor cells (Fig. 2C). One tumor displayed myxoid stroma, in which tumor cells containing clear cytoplasm and eosinophilic tumor cells were interspersed (Fig. 2B), morphologically resembling myoepithelial carcinoma. Immunostaining showed that this tumor was negative for S100, SOX10, smooth muscle actin, and p63, arguing against myoepithelial carcinoma. The other eosinophilic tumor contained lumina but lacked definitive glandular differentiation. Mucicarmine and PAS special staining were not performed. This case was originally diagnosed as poorly differentiated adenosquamous cell carcinoma.

**Fig. 3:**
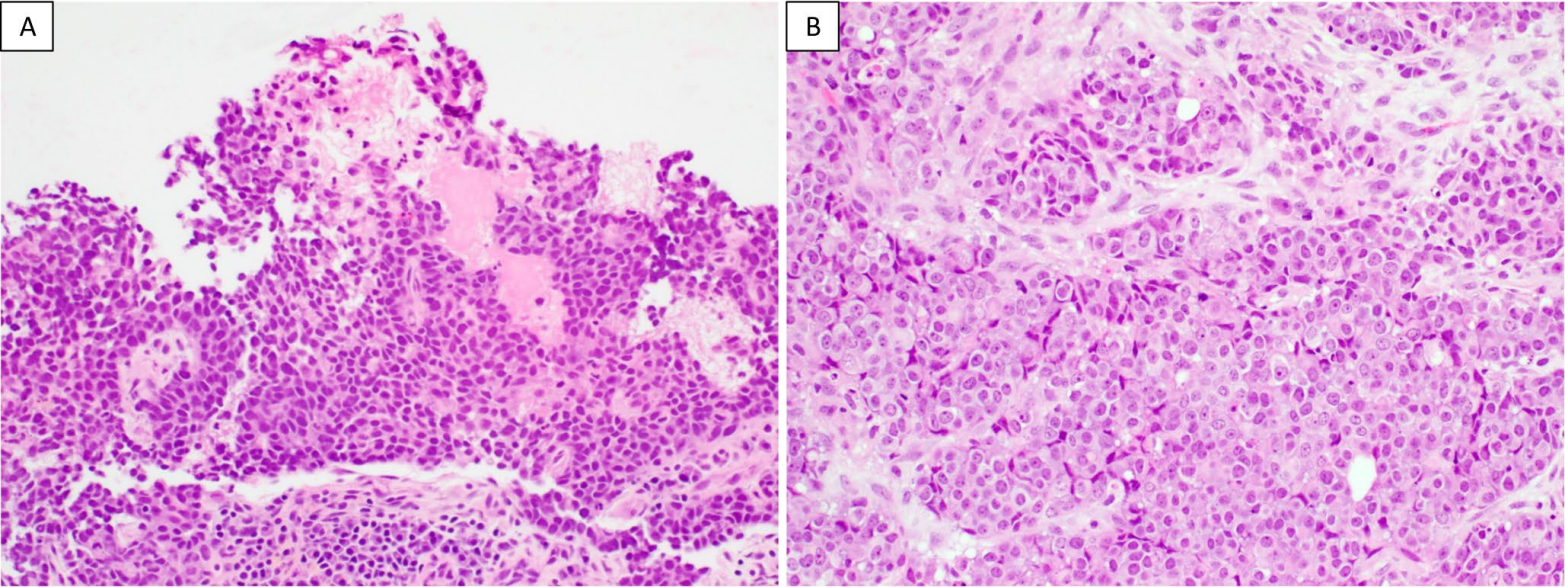
A SMACRB1-dificient sinonasal carcinoma with mixed basaloid and eosinophilic tumor morphology. **A.** The basaloid tumor is predominantly located on the surface, forming exophytic/papillary architecture (200x). **B**. The eosinophilic tumor cells, with prominent plasmacytoid morphology, infiltrate the stroma with associated desmoplastic changes. Images are from case 5

The final case had mixed morphology, with a predominantly basaloid component and approximately 30% of the tumor composed of eosinophilic tumor cells. The basaloid component formed exophytic/papillary structures on the surface (Fig. 3A) and infiltrated into the stroma. The eosinophilic component exhibited infiltrative growth with stromal desmoplasia (Fig. 3B). The nuclear morphology was monotonous and similar in both components.

The immunophenotype of the SMARCB1-deficient sinonasal carcinoma is summarized in Table 2. Cytokeratin expression, demonstrated by pancytokeratin, CK5/6 or CK7, was present in 6/6 cases tested. P63/p40 expression was observed in 4/6 cases. Synaptophysin/chromogranin expression was variably observed in 4/6 cases. One tumor expressed TTF1 and CDX2 (focal). Epstein-Barr virus ISH was negative in 3/3 cases. NUT expression was absent in all 7 cases.

**Table 2:** Immunophenotype of the SMARCB1-deficient sinonasal carcinoma

Case	AE1/3	CK5/6	CK7	p63	p40	CD99	desmin	SYN	Chro	S100	SOX10	CD117	TTF1	CDX2	NUT	p16	HPV	EBER	BRG1	INI1
1	+					-		-							-	+/-*	-		Intact	Loss
2	+				+			+	-						-	+/-*	-		Intact	Loss
3		+	-	+				-	-						-	-	-		Intact	Loss
4	+	+	-	+	+	-	-	+	-	-		-			-	+/-*	-	-	Intact	Loss
5					+			+		-					-	+/-*	-	-	Intact	Loss
6	+	-	-		-		-	+	+	-	-		+	+	-	-	-	-	Intact	Loss
7	+	-	+	-	-									-	-	+/-*	-		Intact	Loss

Syn, synaptophysin; Chro, chromogranin; p16 +/-* indicates aberrant nuclear pronounced p16 staining pattern

A distinctive pattern of p16 expression was observed in 5/7 SMARCB1-deficient sinonasal carcinomas. These tumors displayed multifocal p16 immunostaining, with staining more pronounced in the nuclei than in the cytoplasm (Fig. 1D and E). This pattern contrasted with the strong nuclear and cytoplasmic p16 staining typically observed in the high-risk HPV-associated carcinoma. Two tumors (one basaloid and one eosinophilic tumor) were negative for p16 expression. HPV E6/E7 ISH testing was negative in all 7 cases (Table 2).

### Genetic Alterations of the SMARCB1 and EWSR1 Genes in the SMARCB1-Deficient Sinonasal Carcinoma

One SMARCB1-deficient sinonasal carcinoma failed the DNA quality control testing, while SNP array analysis was successfully performed on the remaining 6 cases. The results are summarized in Table 3. Four of six (67%) cases demonstrated homozygous *SMARCB1* loss, while two cases (33%) demonstrated heterozygous *SMARCB1* loss.

**Table 3:** Genetic alterations on chromosome 22q encompassing *SMARCB1* and *EWSR1* assessed by SNP array and *EWSR1* gene FISH analysis

Case	SMARCB1 by SNP	EWSR1 by SNP	EWSR1 FISH *	Regions and size involved by deletion or LOH by SNP	Other neighboring gene alteration
1	Failed	Failed	Positive	N/A	N/A
2	Homozygous deletion	Heterozygous deletion	Positive	22q11.23q12.2 (8.6 Mb)	*MN1*,* CHEK2*,* NF2*,* PATZ1*
3	Homozygous deletion	LOH	Negative	22q11.21q13.33 (30.2 Mb)	*LZTR1*,* MAPK1*,* IGL*,* BCR*,* MN1*,* CHEK2*,* NF2*,* PATZ1*,* MYH9*,* APOBEC3B*,* PDGFB*,* MRTFA*,* EP300*
4	Heterozygous deletion	Intact	Negative	22q11.23 (1.8 Mb)	N/A
5	Homozygous deletion	Heterozygous deletion	Positive	22q11.21q12.3 (11.4 Mb)	*MAPK1*,* IGL*,* BCR*,* MN1*,* CHEK2*,* NF2*,* PATZ1*
6	Homozygous deletion	Heterozygous deletion	Positive	22q11.21q12.2 (10.1 Mb)	*MAPK1*,* IGL*,* BCR*,* MN1*,* CHEK2*,* NF2*
7	Heterozygous loss (loss of entire 22q)	Heterozygous loss (loss of entire 22q)	Positive	Entire 22q	Entire 22q

*EWSR1 FISH: “Positive” indicates presence of heterozygous *EWSR1* gene loss. “Negative” indicated no abnormality

LOH, loss of heterozygosity

**Fig. 4:**
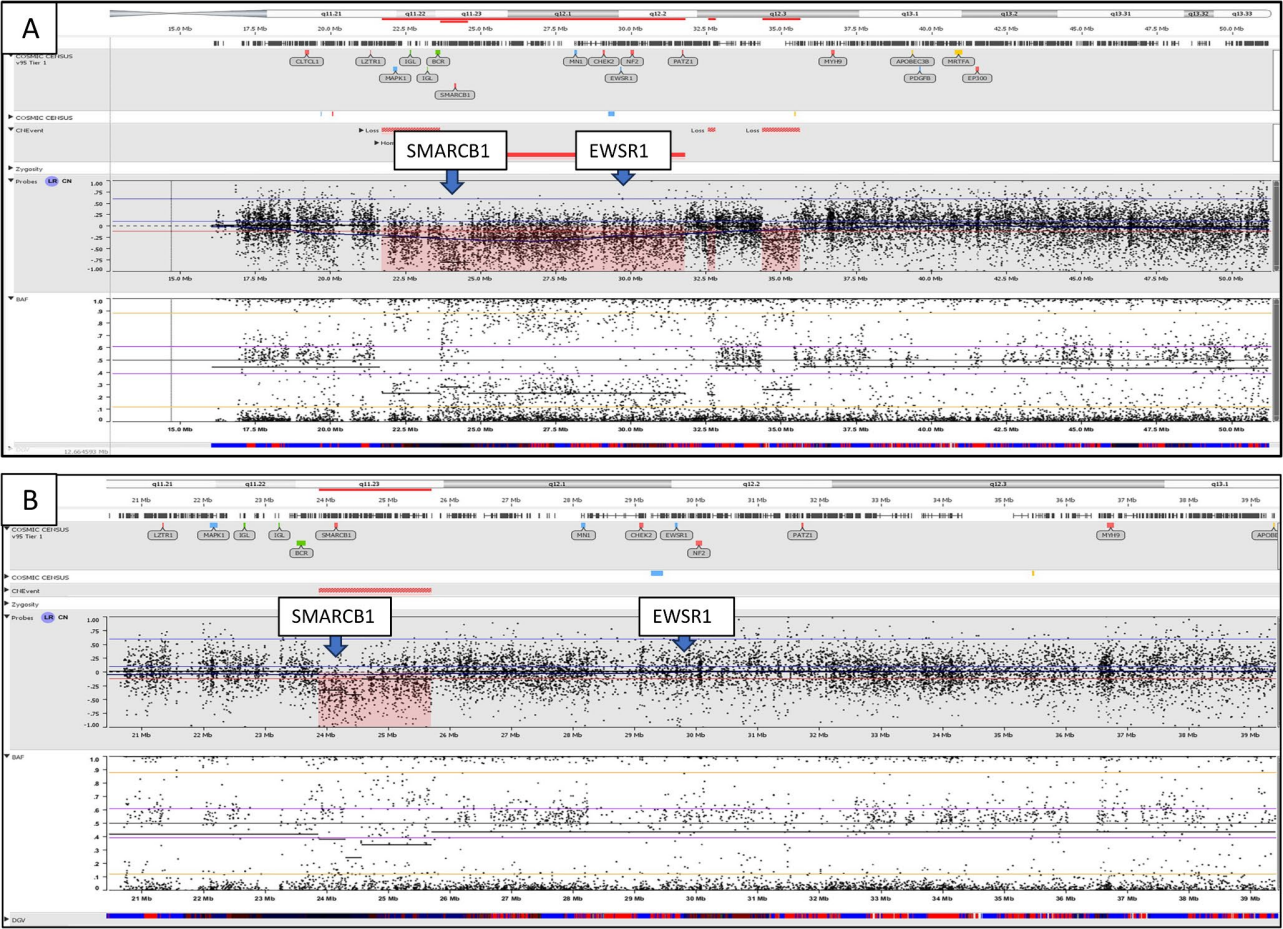
Representative images of single nucleotide polymorphism (SNP) array analysis targeting *SMARCB1* and *EWSR1* (22q11.23–q12.2). **A**. This case (case 6) shows a 10.1-megabase deletion of chromosome 22q11.21-q12.2. The proximal 922-kilobase portion of the deletion is homozygous and contains the *SMARCB1* gene. The *EWSR1* gene is a part of distal heterozygous deletion. Two additional heterozygous deletions of 22q12.3 with no COSMIC census Tier 1 genes are present. **B**. This case (case 4) shows a 1.8-megabase heterozygous deletion of chromosome 22q11.23 containing the *SMARCB1* gene, while the *EWSR1* gene remains intact

Five cases exhibited complex genetic alterations. A heterozygous loss of the entire chromosome 22q was observed in one tumor (Case 7). A large deletion spanning 22q11.21q12.3, ranging from approximately 8.6 to 11.4 megabases, was detected in three tumors (Cases 2, 5, and 6). This deletion included a homozygous deletion of the *SMARCB1* gene and a distal heterozygous deletion encompassing the *EWSR1* gene (Fig. 4A). A 30.2-megabase loss of heterozygosity (LOH) in 22q11.21q13.3, along with a nested 982.3-kilobase homozygous deletion that included the *SMARCB1* gene, was identified in Case 3. Of note, Case 5 underwent STRATA testing, which revealed a deep deletion of the *SMARCB1* gene. Neighboring genes centromeric/proximal to the *SMARCB1*, such as *MAPK1*,* IGL*, and *BCR*, as well as genes telomeric/distal to the *EWSR1* gene, such as *NF2* and *PATZ1*, were variably affected by the large deletion on chromosome 22q. Case 4 exhibited a 1.8-megabase heterozygous deletion of chromosome 22q11.23, affecting only the *SMARCB1* gene (Fig. 4B).

**Fig. 5:**
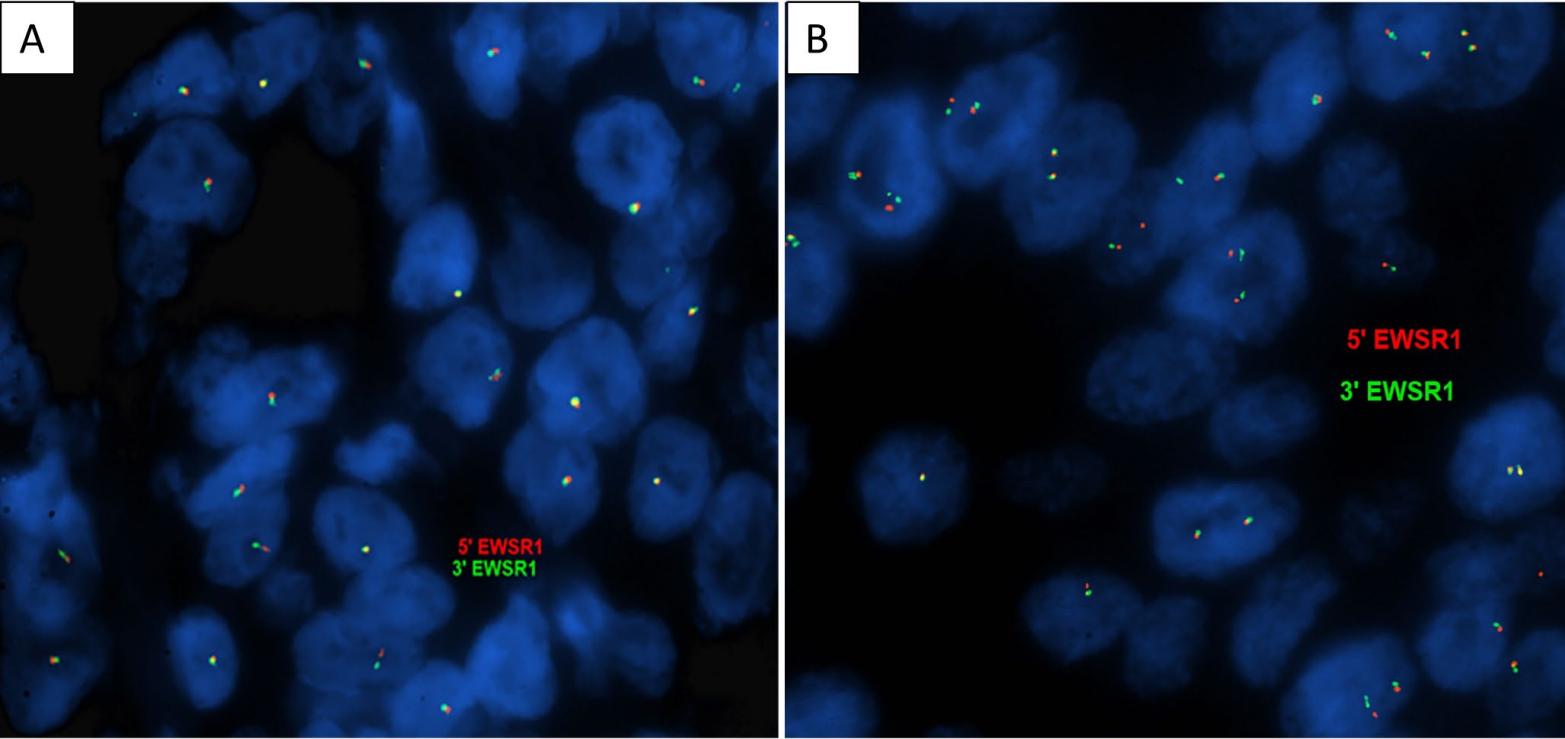
Representative images of the *EWSR1* gene FISH assay. The *EWSR1* gene FISH assay demonstrates heterozygous loss of the *EWSR1* gene in case 6 (**A**) and intact *EWSR1* gene copies in case 4 (**B**)

*EWSR1* gene FISH analysis was successfully conducted on all SMARCB1-deficient sinonasal carcinomas (summarized in Table 3). Heterozygous *EWSR1* gene loss was observed in 5/7 tumors (Fig. 5A) while two tumors (Cases 3 and 4) did not show *EWSR1* copy loss by FISH (Fig. 5B). None of the tumors showed *EWSR1* gene rearrangement. The *EWSR1* gene FISH results were consistent with the SNP array findings.

There was no correlation between tumor morphology and the *SMARCB1/EWSR1* gene alteration patterns.

## Discussion

SMARCB1-deficient sinonasal carcinoma is a rare tumor, with fewer than 200 cases reported [11, 15]. It is possible that many such tumors have been misclassified as other tumor types. The reported frequency of SMARCB1-deficient sinonasal carcinomas ranges from 2 to 7%, based on a small number of systematic cohort studies [12, 16, 17]. SMARCB1-deficient sinonasal carcinomas constitute 4.7% of sinonasal carcinomas in our cohort; and none were SMARCA4-deficient carcinomas.

All seven SMARCB1-deficient sinonasal carcinoma patients were male. This finding aligns with the slight male predominance reported in the literature, with a male-to-female ratio of 1.6, based on a systematic review of 128 patients [11]. All tumors in our cohort primarily arose in the paranasal sinuses (4 in the maxillary sinus and 3 in the ethmoid sinus). Previously reported cases also have shown that these tumors predominantly involved the paranasal sinuses, with the ethmoid sinus being the most commonly affected [15]. The clinicopathologic data from our study reiterate the aggressive behavior of the SMARCB1-deficient sinonasal carcinoma.

Our study re-illustrates the wide morphologic and immunophenotypic spectrum of the SMARCB1-deficient sinonasal carcinoma. The majority of SMARCB1-deficient sinonasal carcinomas exhibited basaloid morphology, with many displaying an exophytic component, almost indistinguishable from the sinonasal nonkeratinizing SCC. Tumors with eosinophilic or pink tumor cells appeared to be more infiltrative morphologically. A correlation between the morphologic subtypes and clinicopathologic parameters and/or patient outcomes cannot be established from the limited number of cases. Such a correlation has not been reported in the literature and could be an interesting subject for future study. Associations between the tumor morphology and patient characteristics, such as the patient age and gender, reported in the literature [21], were not observed in our study.

Immunophenotypically, a subset of SMARCB1-deficient sinonasal carcinomas exhibited features that overlap with the SCC, and some tumors expressed neuroendocrine markers. One tumor expressed TTF-1 and CDX2. The diverse immunophenotypes increased diagnostic challenges. In fact, a definitive diagnosis of SMARCB1-deficient sinonasal carcinoma was not established in the initial biopsy specimens from two cases (Cases 2 and 6); an accurate diagnosis was rendered in the resection specimens. Expression of CDX2 and hepatocyte-specific antigen has been reported in the SMARCB1-deficient sinonasal carcinoma previously [21]. Caution should be exercised when interpreting the expression of lineage-specific markers, such as TTF1 and CDX2, in poorly differentiated sinonasal carcinomas to avoid misclassification.

A subset of the SMARCB1-deficient sinonasal carcinoma has been reported to be p16 positive in the literature [12, 16]. We observed a distinct p16 expression pattern with pronounced nuclear staining in 5/7 tumors, while none exhibited the strong nuclear and cytoplasmic staining - the block-pattern immunoreactivity– typically observed in HPV-associated carcinomas.

Molecular and genomic studies performed on a subset of SMARCB1-deficient sinonasal carcinomas using FISH or next-generation sequencing have revealed complex molecular alterations underlying SMARCB1 protein loss. Homozygous deletion of the *SMARCB1* gene was found in the majority of cases while heterozygous deletion was also observed, yet with less frequency [12, 20, 21, 26]. A large gene panel analysis, including *TP53*,* CTNNB1*, and *IDH2*, revealed no additional oncogenic mutations, supporting SMARCB1 protein loss as the primary driver of this distinct carcinoma in the sinonasal tract [21, 27].

Genetic studies from our cohort, focusing on a large gene fragment of chromosome 22q encompassing the *SMARCB1* and neighboring genes, revealed homozygous deletion of the *SMARCB1* gene in 4/6 cases and heterozygous *SMARCB1* gene deletion in remining 2 cases. Additional mechanisms related to SMARCB1 protein loss in the two tumors harboring heterozygous *SMARCB1* gene deletion could include mutations and/or epigenetic silencing, which were not explored in this study, representing a limitation of our research.

Extended gene alterations flanking the *SMARCB1* gene (22q11.23) on both the centromeric and telomeric sides was identified in 5/6 cases, including heterozygous loss of the entire chromosome 22q in one case, heterozygous *EWSR1* gene deletion in 3 cases and loss of heterozygosity (LOH) in one case. Extended copy number loss of genes neighboring *SMARCB1* has been reported in other SMARCB1-deficient mesenchymal tumors [19, 28] and SMARCB1-deficient sinonasal carcinoma as well [21]. A comprehensive targeted next-generation sequencing study on a relatively large cohort of the SMARCB1-deficient sinonasal carcinoma (*n* = 22) revealed the loss of at least one *SMARCB1* allele, with most cases (13/19, 68%) showing homozygous deletion [21]. Additionally, 6 out of 12 cases from the same study showed concurrent losses of genes in close proximity to *SMARCB1* on 22q, affecting both centromeric and telomeric regions [21], similar to our findings. Alteration of the *EWSR1* gene was not reported in this study. Loss of the entire chromosome 22q has been reported in other human tumors [29–31] but has not yet been documented in SMARCB1-deficient sinonasal carcinomas.

SMARCB1-deficient sinonasal carcinomas account for 4.7% of carcinomas of the sinonasal tract in this single-institution cohort, while no cases of SMARCA4-deficient sinonasal carcinoma were identified. SMARCB1-deficient sinonasal carcinomas exhibit a broad morphologic and immunohistochemical spectrum, underscoring the importance of performing relevant INI1 and BRG1 immunohistochemical staining when classifying sinonasal tumors. The majority of SMARCB1-deficient sinonasal carcinomas demonstrate homozygous deletion of the *SMARCB1* gene and frequently harbor gene loss flanking the *SMARCB1* gene, including the *EWSR1* gene. Recognizing the secondary *EWSR1* gene abnormalities in SMARCB1-deficient tumors can avoid misinterpretation of *EWSR1* gene FISH assay and misclassification of the tumor.

## Electronic Supplementary Material

Below is the link to the electronic supplementary material.

Supplementary Material 1

## Data Availability

The data that support the findings of this study are not openly available due to reasons of sensitivity and are available from the corresponding author upon reasonable request.
