# Supplementary material for: Clinicopathologic and Molecular Characterization of SMARCB1-Deficient Sinonasal Carcinomas – A Systematic Study from a Single Institution Cohort
Source: Head Neck Pathol. 2025 May 14;19(1):60. doi: 10.1007/s12105-025-01788-w (PMC12078905; doi:10.1007/s12105-025-01788-w)
Supplement: Supplementary file 1 — Supplementary Material 1 [file 12105_2025_1788_MOESM1_ESM.docx]

**Supplemental Table 1.** The Antibodies and the probe used for immunohistochemical staining and in situ hybridization performed during the initial workup of the cases

| **Antibody or Probe** | **Clone or Kit** | **Manufacture/Vendor Company** |
| --- | --- | --- |
| AE1/3 | AE1/AE3 | DAKO |
| CK5/6 | D5/16B4 | Roche |
| CK7 | SP9 | Roche |
| P63 | BC4A4 | BIOCARE |
| P40 | BC28 | Roche |
| CD99 | EPR30974 | CELL MARQUE |
| Desmin | DE-R-11 | Roche |
| Synaptophysin | SP11 | Roche |
| Chromogranin | LK2H10 | Roche |
| S100 | 4C4-9 | Roche |
| SOX10 | SP267 | Roche |
| CD117 | YR145 | CELL MARQUE |
| TTF1 | SPT24 | BIOCARE |
| CDX2 | EPR2764Y | Roche |
| EBER probe | INFORM EBER PROBE-ISH | Roche |
